# Elevated temperatures are associated with stress in rooftop-nesting Common Nighthawk (*Chordeiles minor*) chicks

**DOI:** 10.1093/conphys/coy010

**Published:** 2018-03-01

**Authors:** Gretchen N Newberry, David L Swanson

**Affiliations:** Department of Biology, University of South Dakota, 414 E Clark ST, Vermillion, SD 57069, USA

**Keywords:** Corticosterone, Common Nighthawk, nestling condition, micro-climate, regional climate, stress response

## Abstract

Grasslands and riparian forests in southeastern South Dakota have been greatly reduced since historical times, primarily due to conversion to row-crop agriculture. Common Nighthawk (*Chordeiles minor*) nesting habitat includes grasslands, open woodlands and urban rooftops, but nesting sites in southeastern South Dakota are confined to rooftops, as natural nesting habitat is limited. Nighthawks nesting on exposed rooftop habitats may encounter thermal conditions that increase operative temperatures relative to vegetated land cover types. Mean humidity has increased and mean wind speed and cloud cover have decreased during the nighthawk breeding season from 1948 to 2016 in southeastern South Dakota. These changes might contribute to increasing operative temperatures at exposed rooftop nest sites and this could influence chick condition. We studied nest micro-climate and the plasma stress response for 24 rooftop-nesting nighthawk chicks from 17 nests during 2015 and 2016. High humidity prior to blood collection reduced both baseline and stress-induced plasma corticosterone (CORT). In contrast, high maximum temperatures during the day before sampling increased stress-induced CORT. The magnitude of the chick stress response was significantly negatively related to maximum wind speed for the week prior to CORT measurement. Other weather and micro-climate variables were not significant effectors of CORT metrics. Most chicks had low baseline CORT and were able to mount a stress response, but a subset of chicks (*n* = 4) showed elevated baseline CORT and a negative association between the magnitude of stress response and ambient temperature. For this subset, mean ambient temperature for the day before sampling was significantly higher (2.3°C) than for chicks with typical baseline CORT levels. These data suggest that regional climate change trends could affect the ability of nighthawk chicks to mount a stress response, which, in turn, might influence the susceptibility of nighthawk chicks to climate change in the Northern Prairie region.

## Teaser

Common Nighthawk (*Chordeiles minor*) nesting habitat is limited to urban rooftops in southeastern South Dakota. Regional climate has changed from 1948 to 2016, with increases in humidity and solar radiation. Chicks exposed to high temperatures showed high baseline plasma corticosterone and low stress responses, suggesting that current climate influences stress response capacity.

## Introduction

Since the 1980s, aerial insectivores have experienced among the most prominent population declines of any bird guild (Sauer *et al.*, 2007; Nebel *et al*., 2010). Common Nighthawks are one such aerial insectivore and have a wide breeding distribution in North America, yet are subject to local population declines. North American Breeding Bird Survey data from 1980 to 2015 show a –1.5% annual population decline for nighthawks in South Dakota (Sauer *et al.*, 2017). This population decline is coincident with a period of agricultural intensification (Wright and Wimberly, 2013) and warming winters that influence bird migration phenology (Swanson and Palmer, 2009) in the Northern Prairie region.

Due to changing land use practices, natural nighthawk nesting sites (i.e. open woodlands and grasslands) in the Northern Prairie region are in decline (Tallman *et al.*, 2002; Wright and Wimberly, 2013). The study area, southeastern South Dakota, is currently dominated by row-crop agriculture, but was historically covered by grasslands (Spess Jackson *et al.*, 1996; Tallman *et al.*, 2002). Such conversion and loss of natural habitat is likely driving greater use of flat, gravel rooftops in urban areas for nesting by nighthawks (Brigham, 1989) and may contribute to population declines for this species. Nighthawk occupancy of exposed urban rooftop nesting sites is affected by rooftop characteristics, including thermal micro-climates (Viel, 2014). Because of higher wind speeds at rooftop than at ground sites, the convective advantages of rooftop sites might help mitigate the higher operative temperatures experienced at exposed rooftop sites compared to ground nest sites (Fisher *et al.*, 2004; Fletcher *et al.*, 2012). However, the characteristics of the parapet surrounding rooftop nest sites may also affect micro-climates and nest success, but not in a straightforward manner, as a trade-off between thermal micro-climate and falling danger likely exists. High parapets on rooftops will likely reduce wind movement resulting in warmer thermal micro-climates at the nest.

Urban habitats often function as ‘heat islands,’ with modified local climate due to reduced vegetation cover, impervious surfaces, and a high density of buildings, which lower evaporative cooling, store heat, and warm the surface air (Bonan, 2002). Exposed urban rooftop nest sites thus have the potential to produce thermally unfavorable conditions for nesting nighthawks, but no recent studies have examined nest micro-climates at urban rooftop nesting sites. Rooftop nesting micro-climates are typically warmer than micro-climates at natural sites, such as grasslands (Weller, 1958; Marzilli, 1989; Lohnes, 2010). If nighthawks are displaced to urban sites and climate change produces micro-climate temperatures that negatively impact nesting success at urban sites, this could negatively impact nighthawk populations, and leave them with few alternatives due to the reduction of natural nest site availability because of anthropogenic habitat disturbance.

Habitats can affect adult reproductive condition by influencing, among other traits, reproductive output, body condition, and the immune system (Wingfield *et al.*, 1992, 1997). Perturbation of habitat can produce elevated baseline levels of corticosterone (CORT, the primary stress hormone in birds) and negatively impact nesting success in birds, although these relationships may be complex (Lucas *et al.*, 2006; Bonier *et al.*, 2007; Schoech *et al.*, 2009). Moreover, elevated baseline corticosterone can also activate immunosuppressants in egg-laying females, which can be incorporated into the yolk, thereby impacting condition of the nestlings (Love *et al.*, 2005) as well as the adults. Habitats also affect micro-climates experienced by breeding birds, which in turn impact physiological condition, including the stress response, which may be suppressed during the hottest days in some species (Wingfield *et al.*, 1992). In addition, short-term temperature changes may be associated with increased baseline plasma corticosterone concentrations in some birds, but not others, especially in those species not specifically adapted to high temperatures (El-Halawani *et al.*, 1973; Xie *et al.*, 2017). Indeed, baseline CORT levels in nestling Common Nighthawks are sometimes positively correlated with exposure to high ambient nest temperatures (Lohnes, 2010). Such exposure could potentially lead to reduced chick survival and fitness by downregulating the immune system or mobilizing energy stores (Sapolosky *et al.*, 2000; Love *et al.*, 2005).

Exposed rooftop nesting sites with high thermal loads have the potential to become unfavorable for nighthawks if climate change produces even higher operative temperatures (Fletcher *et al.*, 2012), particularly in urban areas as a result of the ‘heat island’ effect. Mean temperatures in the Great Plains are expected to increase by 3.6–6.1°C over the next 100 years (Ojima and Lackett, 2002). For example, mean summer temperatures and mean summer dew points at Sioux City, Iowa (an urban area and the nearest weather station to some of our field sites), are expected to increase by 6.3°C and 1.1°C, respectively, by the year 2100 (Kenward *et al.*, 2014). As a result of this climate change, breeding ranges for Great Plains bird species are expected to be reduced by up to 35% (Peterson, 2003).

If sufficient natural nesting sites are available within a region, nighthawks prefer natural nesting sites to urban rooftops, suggesting that natural sites might provide better nesting conditions than rooftop sites (Brigham, 1989). If urban rooftop habitats provide lower-quality nesting habitat than natural habitats, then this may manifest as higher baseline plasma corticosterone (CORT_B_) levels and a reduced stress response for birds using rooftop habitats with hotter micro-climates. While adult Caprimulgids tolerate hot temperatures (Cowles and Dawson, 1951; Howell, 1959; Bartholomew *et al.*, 1962; Lasiewski and Dawson, 1964; O’Connor *et al.*, 2017), nest micro-climates that minimize heat loss in cold weather and maximize heat loss during higher temperatures are generally important to Caprimulgids (Kortner and Geiser, 1999). This may be particularly true for chicks, with less developed thermoregulatory capacities than adults. Thus, favorable micro-climates at nest sites for Common Nighthawks are likely to be important to nesting success.

In this study, we evaluated nest conditions (i.e. micro-climate) and plasma corticosterone of chicks at rooftop nest sites in southeastern South Dakota and examined correlations of these traits with a variety of climatic variables to determine which climate variables are most associated with CORT levels in nighthawk chicks. We hypothesized that baseline CORT levels of chicks will be positively associated with the maximum operative temperature (*T*_e_) encountered at the nest and to the duration of exposure to hot operative temperatures.

## Material and methods

### Nest searches

**Figure 1:**
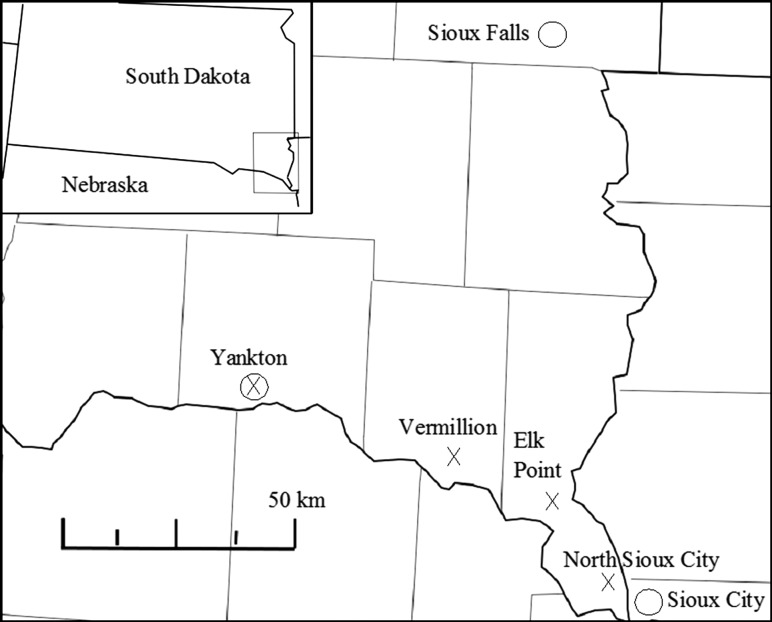
Cities with rooftop nest study sites (i.e. X) and NOAA weather stations (i.e. Circles) in South Dakota and Iowa.

One to three months prior to the nesting season, we contacted property managers of 32 school, hospital and storefront rooftops in North Sioux City, Elk Point, Vermillion and Yankton, South Dakota (Fig. 1), and received permission to access 22 of them. Several property managers required an agreement indemnifying the property owner prior to granting rooftop access permission. We conducted systematic nest searches on these rooftops by laying out a grid network with 1 m × 1 m squares on graveled areas of the rooftop and walking all gridlines until adult birds flushed. When we flushed adults, we carefully searched the area where the adult flushed for eggs or chicks.

### Blood collection and corticosterone measurement

For CORT measurements, we collected blood samples (<100 µl) from 7- to 14-day-old chicks from the brachial vein (Wingfield *et al.*, 1994) in heparinized capillary tubes. All blood samples were collected within 3 min after the female flushed from the nest. Following the first blood sample, we placed individual chicks in cloth bags in a shaded location for 30 min, after which we collected a second blood sample for measurement of the stress response (i.e. increase in corticosterone following handling stress) (Wingfield *et al.*, 1994). We stored blood samples on ice in microcentrifuge tubes while in the field. Following blood collection, we banded chicks with a standard USFWS aluminum leg band, performed morphometric measurements, and released them back to their nest site. For morphometrics, we measured unflattened wing chord with calipers to the nearest 0.1 mm and measured mass to the nearest g with a Pesola spring balance.

Upon return to the laboratory, we centrifuged blood samples for 10 min at 3000 × *g* at 4°C, drew off the plasma, and stored plasma frozen at −70°C until later analyses. We assayed plasma CORT with commercially available spectrophotometric kits (ELISA kits ADI-900-097), as previously conducted in our laboratory (Liu and Swanson, 2014). We sampled 24 nestlings from 17 nests during the 2015 and 2016 breeding seasons.

### Nest micro-climate

We collected nest micro-climate data using two methods. First, we placed iButton data loggers (DS1921G-F5# Thermochron) in the nest scrape next to the eggs or chicks and, once chicks hatched, moved them every week to where the chicks had relocated to record ambient ‘nest’ temperature (*T*_a_). We programmed the iButtons to record nest temperature (°C) every 10 min from incubation to fledging (Ardia *et al.*, 2006). Second, we deployed operative temperature (*T*_e_) thermometers within 1–2 m of the eggs or chicks at sites with similar exposure conditions to the nest site (e.g. shade prevalence and amount of gravel substrate) and, after hatching, moved them every week to where the chicks had relocated. This distance from the nest site was far enough away to avoid disturbing the birds (G. Newberry unpublished data). We designed operative temperature (*T*_e_) thermometers using 10 × 12 cm copper ovoid toilet floats (approximately the same volume as an adult nighthawk) with the outside surface painted flat gray. We cut a 2.5-cm hole in the copper ovoid to attach a 2-m Cu–Cn thermocouple probe connected to a Model UX120006M 4-channel analog input HOBO data logger (Hobo Instruments, Contoocook, NH), which we placed inside the copper ovoid. We recorded operative temperature once per minute from egg discovery date to chick fledging. During weekly nest visits, we also placed a CIH20DL Data Logging Hot Wire Anemometer and infrared thermometer (General Tools & Instruments, Secaucus, NJ) adjacent to the chicks or eggs for 30 s to record maximum wind speed (KPH) and maximum temperature to allow periodic estimation of the potential for convective heat loss.

### Regional climate & roof characteristics

We collected 1948–2016 regional climate data for the three weather stations (Yankton and Sioux Falls, South Dakota, and Sioux City, Iowa; NOAA, 1948–2016) nearest to our study sites (Fig. 1) during the Common Nighthawk breeding season (15 May–15 August). We used hourly temperature (°C), relative humidity (%), dew point (°C), maximum wind gust (KPH), precipitation (cm), cloud cover (%) and wind speed (KPH) to calculate mean daily and weekly maximums for each metric prior to blood sampling. We calculated the roof height (m) with a Bushnell Yardage Pro Sport 450 laser rangefinder. We measured the height of the roof parapet with a tape measure or calipers to the nearest mm.

### Statistics

To evaluate how temperature changed over time, we performed simple linear regression analyses of breeding season (15 May 15–August) means of maximum, minimum and average daily temperatures from regional climate recordings for 1948–2016 using the R 3.3.2 car package (Fox and Weisberg, 2011) with year as the predictor variable. To analyze how plasma CORT measurements changed with micro-climate, regional climate, rooftop and morphometric variables, we used multiple regression of CORT measurements as a function of these independent variables. We performed multiple regressions with three response variables, CORT_B_, stress-induced CORT (CORT_30_) and the magnitude of the stress response (CORT_30_–CORT_B_). We log_10_-transformed the residuals for all CORT variables (log[CORT/Mean+1]) to meet the assumptions of normality and equal variance. For cases where the stress response was ≤0 (10 out of 24 samples), we assigned a value of 1 for the magnitude of the stress response.

We used five different multiple regression models for these analyses: temporal (i.e. Year, Julian Date, Julian Date by Year interaction term, Time of Day [hours since midnight]), rooftop conditions (i.e. roof parapet height [cm] above roof surface, roof height [m] above ground), chick condition at blood collection (i.e. mass [g], wing chord [cm]), nest micro-climate (i.e. maximum anemometer temperature [°C] and wind speed [KPH] at blood collection, iButton temperature [°C] at blood collection, operative temperature [*T*_e_, °C] at blood collection), and regional (weather station) climate variables. For regional climate variables, we calculated maximum daily temperature (°C), maximum daily dew point (°C), mean daily maximum dew point (°C), maximum relative humidity (%), maximum wind speed (KPH), mean daily maximum wind speed (KPH), and mean daily maximum relative humidity (%) for 24-h and 7-day periods prior to blood sampling from the weather station (i.e. in Yankton, South Dakota or Sioux City, Iowa) nearest to the nest.

Because chick CORT_B_ levels fell into two non-overlapping groups (CORT_B_ < 25 ng/ml, *n* = 20; and CORT_B_ > 50 ng/ml, *n* = 4), we compiled these five models for all chicks combined and for each group separately. Our total sample size was 24, but for all of these analyses, where there were nests with missing data due to equipment malfunction (i.e. *n* = 1 for roof characteristics, *n* = 2 for anemometer *T*_a_ and wind speed, *n* = 4 for *T*_e_, *n* = 15 for iButton *T*_a_, as indicated in Tables 1–3 and S1–S2), thus invalidating the multiple regression approach, we performed simple linear regressions of predictor variables against plasma CORT. For any variables that were associated with CORT metrics in low or high CORT_B_ groups but not in the other, to compare mean values between the two groups, we also performed two-tailed two-sample Welch’s *t*-tests because the variances were unequal.

## Results

Mean humidity and maximum wind speed increased significantly and cloud cover and mean wind speed decreased significantly over the 1948–2016 period for two of the three weather stations (Table 1). The only rooftop characteristic significantly associated with micro-climate variables was roof height, which was significantly positively correlated with iButton temperature (Table S1). However, this result was associated with a small sample size and can be explained by one particularly high and hot rooftop (Table S1). Removing this point from the regression eliminated the significant result.

**Table 1:** Summary of regression analyses of interval means of daily summer (15 May–15 August, 1948–2016) environmental data (as dependent variables) from three surrounding weather stations (Yankton, SD, Sioux City, IA, and Sioux Falls, SD) with year as a predictor variable

Station	Dependent variable	*F*	*df*	Adj *r*^2^	*P*	*Coef*
Yankton	Maximum Temp (°C)	6.04	1,67	0.069	<0.05	−0.022
	Mean Temp (°C)	0.5109	1,66	−0.007	0.48	0.007
	Minimum Temp (°C)	4.204	1,67	0.045	<0.05	0.033
	Maximum Dew Point (°C)	0.0095	1,42	−0.024	0.92	−0.001
	Mean Dew Point (°C)	1.55	1,42	0.013	0.22	−0.024
	Minimum Dew Point (°C)	1.84	1,42	0.019	0.18	−0.033
	Maximum Humidity (%)	14.42	1,42	0.238	<0.001	0.238
	Mean Humidity (%)	13.13	1,42	0.22	<0.001**	0.224
	Minimum Humidity (%)	1.048	1,42	0.001	0.31	−0.070
	Maximum Wind (KPH)	9.305	1,42	0.162	<0.05**	0.139
	Mean Wind (KPH)	0.410	1,42	−0.014	0.53	−0.013
	Precipitation (cm)	0.458	1,52	−0.010	0.50	0.010
	Cloud Cover (%)	112.6	1,42	0.722	<0.001**	−0.091
Sioux City 1	Maximum Temp (°C)	0.363	1,70	−0.009	0.55	0.005
	Mean Temp (°C)	0.1168	1,70	−0.013	0.73	−0.001
	Minimum Temp (°C)	4.402	1,70	0.0457	<0.05	−0.010
	Maximum Dew Point (°C)	1.928	1,70	0.013	0.169	0.007
	Mean Dew Point (°C)	2.085	1,70	0.015	0.15	0.008
	Minimum Dew Point (°C)	1.96	1,70	0.013	0.17	0.008
	Maximum Humidity (%)	2.614	1,70	0.022	0.11	0.036
	Mean Humidity (%)	3.679	1,70	0.036	0.06	0.045
	Minimum Humidity (%)	1.463	1,70	0.006	0.23	0.030
	Maximum Wind (KPH)	3.901	1,70	0.039	0.052	0.033
	Mean Wind (KPH)	11.61	1,70	0.13	<0.001**	−0.030
	Precipitation (cm)	2.632	1,68	0.023	0.109	−0.011
	Cloud Cover (%)	7.817	1,44	0.132	<0.001**	−0.026
Sioux Falls	Maximum Temp (°C)	0.041	1,70	−0.014	0.84	0.002
	Mean Temp (°C)	0.080	1,70	−0.013	0.78	0.002
	Minimum Temp (°C)	0.104	1,70	−0.013	0.75	0.002
	Maximum Dew Point (°C)	7.195	1,70	0.080	<0.001	0.015
	Mean Dew Point (°C)	11.71	1,70	0.131	<0.001	0.020
	Minimum Dew Point (°C)	15.18	1,70	0.167	<0.001	0.025
	Maximum Humidity (%)	3.113	1,70	0.029	0.08	0.039
	Mean Humidity (%)	5.499	1,70	0.060	<0.05**	0.062
	Minimum Humidity (%)	7.096	1,70	0.080	<0.001	0.075
	Maximum Wind (KPH)	4.115	1,70	0.042	<0.05**	0.029
	Mean Wind (KPH)	13.19	1,70	0.147	<0.001**	−0.026
	Precipitation (cm)	0.010	1,67	−0.015	0.922	−0.200
	Cloud Cover (%)	0.060	1,46	−0.020	0.81	0.002

Dependent variables with *P* < 0.05 at same direction of Coefficient at all three weather stations are denoted with *. Dependent variables with *P* < 0.05 at same direction of Coefficient at two weather stations are denoted with **.

Regional (weather station) climate variables were the only significant predictors of CORT metrics for all chicks pooled (Table S2). Baseline CORT was significantly negatively related to maximum dew point for the day of measurement and maximum humidity for the week prior to measurement. In addition, stress-induced CORT was significantly positively related to maximum *T*_a_ on the day of measurement and significantly negatively related to maximum dew point on the day of measurement. The magnitude of the stress response was significantly negatively related to maximum wind speed for the week prior to measurement. None of the other temporal, roof, chick condition, chick number, or other micro-climate or regional climate variables were significant predictors of CORT metrics.

**Figure 2:**
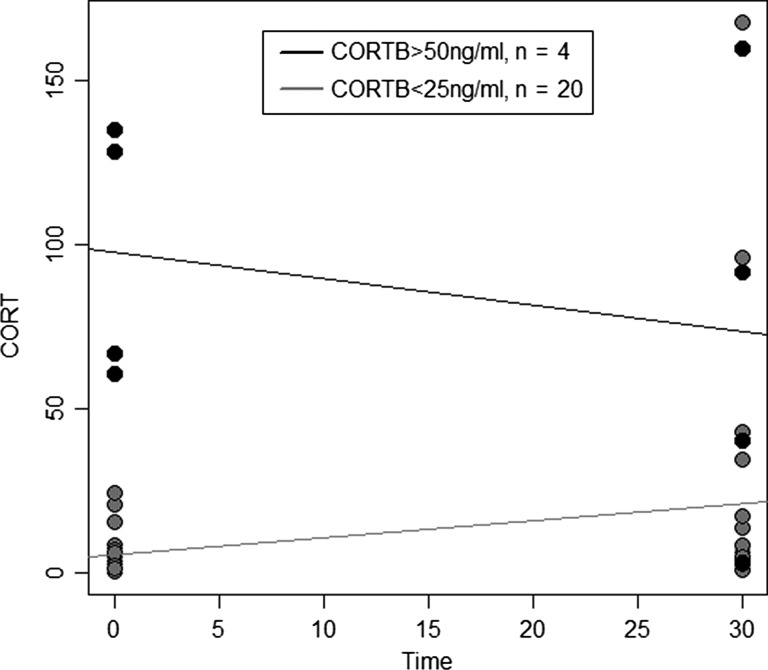
Relationship between corticosterone (CORT) and time of blood sampling.

Because chicks fell into two, non-overlapping groups based on their baseline CORT (Fig. 2), we also analyzed these two groups separately. Mean CORT_B_ was 5.53 ± 1.54 (SE) ng/ml for the low CORT_B_ group and 97.61 ± 19.66 (SE) ng/ml for the high CORT_B_ group. These values differed significantly (Welch’s *t*_3_ = 4.670, *P* = 0.009). The low CORT_B_ group had a mean stress response (CORT_30_–CORT_B_) of 15.40 ± 8.93 (SE) ng/ml, and the high CORT_B_ group had a mean stress response of −24.04 ± 46.05 (SE) ng/ml.

**Figure 3:**
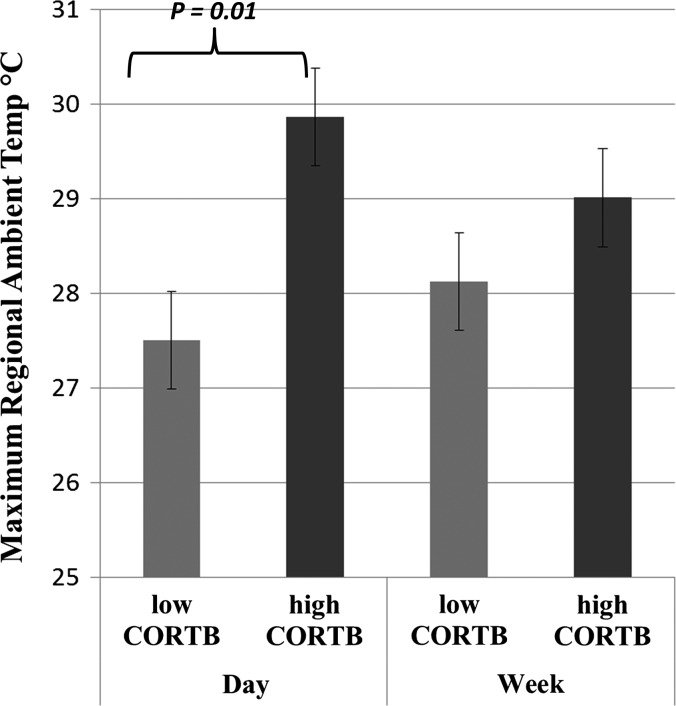
Difference in ambient temperature means between low and high baseline CORT groups.

For the low CORT_B_ group, there were no significant predictors for any CORT metric, except for a year effect on CORT_30_, with lower values in 2016 (Table 2). However, when we compared mean CORT_30_ between the 2 years with a *t*-test for the low CORT_B_ group, there was no significant difference (Welch’s *t*_18_ = 0.682, *P* = 0.51). For the high CORT_B_ group, the only significant result occurred for the magnitude of stress response, where maximum ambient temperature for the day and week prior to capture were both negatively associated with the magnitude of the stress response (Table 3). The maximum temperature for the day prior to blood sampling was significantly higher for the high CORT_B_ group than for the low CORT_B_ group (Welch’s *t*_22_ = −2.744, *P* = 0.01), but the mean maximum temperature for the week prior to blood sampling did not differ significantly between the two groups (Fig. 3).

**Table 2:** Summary of regression analyses of log(CORTB/Mean+1), log(CORT30/Mean+1) and adjusted log(CORT_30_–CORT_B_/Mean+1) (as dependent variables) for chicks with CORT_B_ < 25.00 ng/ml, the low CORT_B_ group (*n* = 20)

Predictor variable model	Predictor variable	Dependent variable	*F*	*df*	Adj *r*^*2*^	*P*	*Coef*
Temporal	Year	CORT_B_	0.474	3,16	−0.091	0.31	−5.947
		CORT_30_	2.873	3,16	0.228	0.02*	−0.015
		CORT_30_–CORT_B_	0.698	3,16	−0.050	0.68	−2.619
	Time of Day	CORT_B_	0.474	3,16	−0.091	0.61	0.013
		CORT_30_	2.873	3,16	0.228	0.55	0.016
		CORT_30_–CORT_B_	0.698	3,16	−0.050	0.63	0.013
Roof Characteristic	Parapet Height (cm)	CORT_B_	2.016	3,15	0.1448	0.45	0.003
		CORT_30_	0.2904	3,16	−0.1341	0.83	−0.001
		CORT_30_–CORT_B_	0.599	3,15	−0.0716	0.61	−0.002
	Roof Height (m)	CORT_B_	2.016	3,15	0.1448	0.05	0.029
		CORT_30_	0.2904	3,16	−0.1341	0.46	0.015
		CORT_30_–CORT_B_	0.599	3,15	−0.0716	0.46	0.012
	Mean Gravel Diameter (cm)	CORT_B_	2.016	3,15	0.1448	0.13	0.019
		CORT_30_	0.2904	3,16	−0.1341	0.52	0.011
		CORT_30_–CORT_B_	0.599	3,15	−0.0716	0.60	−0.007
Chick Condition	Mass (g)	CORT_B_	0.1432	2,17	−0.0999	0.68	0.002
		CORT_30_	0.9971	2,17	−0.0003	0.40	0.004
		CORT_30_–CORT_B_	2.458	2,17	0.1331	0.74	0.001
	Wing Length (cm)	CORT_B_	0.1432	2,17	−0.0999	0.68	0.001
		CORT_30_	0.9971	2,17	−0.0003	0.40	−0.003
		CORT_30_– CORT_B_	2.458	2,17	0.1331	0.06	−0.005
Regional Climate	Maximum *T*_a_ (°C) for Day	CORT_B_	5.000	12,7	0.7163	0.09	−0.283
		CORT_30_	5.743	12,7	0.7497	0.96	−0.010
		CORT_30_–CORT_B_	1.874	12,7	0.3556	0.71	0.094
	Maximum *T*_a_ (°C) for Week	CORT_B_	5.000	12,7	0.7163	0.12	0.150
		CORT_30_	5.743	12,7	0.7497	0.14	0.170
		CORT_30_–CORT_B_	1.874	12,7	0.3556	0.87	0.025
	Maximum Dew Point (°C) for Day	CORT_B_	5.000	12,7	0.7163	0.36	0.179
		CORT_30_	5.743	12,7	0.7497	0.99	0.003
		CORT_30_–CORT_B_	1.874	12,7	0.3556	0.80	0.030
	Maximum Dew Point (°C) for Week	CORT_B_	5.000	12,7	0.7163	0.65	−0.076
		CORT_30_	5.743	12,7	0.7497	0.80	−0.334
		CORT_30_–CORT_B_	1.874	12,7	0.3556	0.61	0.071
	Maximum Humidity (%) for Day	CORT_B_	5.000	12,7	0.7163	0.54	−0.069
		CORT_30_	5.743	12,7	0.7497	0.94	0.010
		CORT_30_–CORTB	1.874	12,7	0.3556	0.85	0.035
	Maximum Humidity (%) for Week	CORT_B_	5.000	12,7	0.7163	0.25	0.282
		CORT_30_	5.743	12,7	0.7497	0.50	0.187
		CORT_30_–CORT_B_	1.874	12,7	0.3556	0.94	0.030
	Maximum Wind (KPH) for Day	CORT_B_	5.000	12,7	0.7163	0.23	0.020
		CORT_30_	5.743	12,7	0.7497	0.42	−0.016
		CORT_30_–CORT_B_	1.874	12,7	0.3556	0.33	−0.027
	Maximum Wind (KPH) for Week	CORT_B_	5.000	12,7	0.7163	0.12	0.075
		CORT_30_	5.743	12,7	0.7497	0.14	−0.083
		CORT_30_–CORT_B_	1.874	12,7	0.3556	0.05	−0.163

Dependent variables with *P* < 0.05 are denoted with *. Variables with no data were not included.

**Table 3:** Summary of regression analyses of log(CORTB/Mean+1), log(CORT30/Mean+1) and adjusted log(CORT_30_–CORT_B_/Mean+1) (as dependent variables) for chicks with CORT_B_ > 50.00 ng/ml, the high CORT_B_ group (*n* = 4)

Predictor variable model	Predictor variable	Dependent variable	*F*	*df*	Adj *r*^*2*^	*P*	*Coef*
Roof Characteristics	Parapet Height (cm)	CORT_B_	0.1832	2,1	−1.196	0.73	−0.0138
		CORT_30_	0.4841	2,1	−0.524	0.52	0.0304
		CORT_30_–CORT_B_	0.2500	2,1	−1	0.93	0.0016
	Roof Height (m)	CORT_B_	0.1832	2,1	−1.196	0.65	−0.3931
		CORT_30_	0.4841	2,1	−0.524	0.54	0.5896
		CORT_30_–CORT_B_	0.2500	2,1	−1	0.67	0.1744
Chick Condition	Mass (g)	CORT_B_	0.1538	2,1	−1.294	0.78	0.0198
		CORT_30_	28.000	2,1	0.9474	0.09	0.0362
		CORT_30_–CORT_B_	0.1916	2,1	−1.169	0.65	−0.0078
	Wing Length (cm)	CORT_B_	0.1538	2,1	−1.294	0.87	−0.0037
		CORT_30_	28.000	2,1	0.9474	0.36	0.0054
		CORT_30_–CORT_B_	0.1916	2,1	−1.169	0.86	−0.0018
Regional Climate	Maximum *T*_a_ (°C) for Day	CORT_B_	4.307	2,1	0.6879	0.23	0.6563
		CORT_30_	0.7602	2,1	−0.1902	0.82	0.1780
		CORT_30_–CORT_B_	1.332 × 10^33^	2,1	1	<0.001*	−4.913 × 10^16^
	Maximum *T*_a_ (°C) for Week	CORT_B_	4.307	2,1	0.6879	0.65	0.0848
		CORT_30_	0.7602	2,1	−0.1902	0.60	−0.2443
		CORT_30_–CORT_B_	1.332 × 10^33^	2,1	1	<0.001*	−1.946 × 10^16^

Dependent variables with *P* < 0.05 are denoted with *. Variables with no data were not included.

## Discussion

Because Common Nighthawks nest in an open scrape on flat, gravel rooftops in our study area, they are relatively exposed to weather and might be expected to be particularly susceptible to climate change. Over the past 68 years, regional climate changes include stable summer temperatures, but increasing humidity, declining cloud cover and decreasing mean wind speed (Kenward *et al.*, 2014). All of these changes could affect the thermal environment used by nighthawks by increasing radiative heat gain, decreasing convective heat loss, and reducing the ability for effective evaporative cooling during the hottest parts of the day. As a consequence, we hypothesized that the thermal environment of the rooftop nest sites might be stressful for nighthawks, including nighthawk chicks. This, in turn, might function to impair chick growth rates and survival at such thermally exposed sites.

The positive relationship between roof height and nest site iButton temperatures in this study offers tentative support for the idea that temperatures increase with roof height, but this relationship was driven by a single outlier rooftop. In addition, roof height was not a significant predictor for any of the CORT metrics in chicks. Thus, rooftop height does not appear to influence nest micro-climate sufficiently to serve as a prominent factor affecting chick condition in rooftop-nesting Common Nighthawks.

Nighthawk chicks in this study fell into two groups based on their baseline CORT metrics. One group showed low (i.e. CORT_B_ < 25 ng/ml, *n* = 20) and the other high (i.e. CORT_B_ > 50 ng/ml, *n* = 4) baseline CORT levels. The low baseline group had CORT_B_ levels (5.53 ± 1.54 (SE) ng/ml) within the range of variation for other semi-precocial chick species. For example, CORT_B_ in 21-day-old Red-legged Kittiwake (*Rissa brevirostris*) chicks was less than 4 ng/ml (Kitaysky *et al.*, 2001) and was less than 5 ng/ml in 4- to 18-day-old Tufted Puffin (*Fratercula cirrhata*) chicks (Kitaysky *et al.*, 2005), but ranged from 10 to 18 ng/ml in 10- to 20-day-old Ring-Billed Gull (*Larus delawarensis*) chicks (Chin *et al.*, 2013). In addition, the low CORT_B_ group in the present study showed a mean stress response of 15.40 ± 8.93 (SE) ng/ml, indicating that these chicks were capable of mounting a stress response. The high baseline CORT group in this study, however, had much higher CORT_B_ levels (97.61 ± 19.66 (SE) ng/ml) and showed a negative association between the stress response and maximum ambient temperatures for the day and week prior to blood sample collection (Table 3). Moreover, mean ambient temperature for the day of sampling was significantly elevated in the high CORT_B_ relative to the low CORT_B_ group (Fig. 3). This suggests that short-term weather might affect both baseline CORT and the ability of some chicks to mount a stress response. Collectively, these data suggest that the majority of our study population does not show a compromised ability to cope with environmental stressors, but for some individual chicks, short-term weather conditions might be thermally stressful. Consistent with this idea, we observed several instances of chick die-offs after heat waves accompanied by high humidity (G.N., personal observation).

Our data highlight some associations between regional climate conditions for the day and week prior to measurement and baseline corticosterone or the magnitude of the stress response (i.e. maximum humidity, wind speed and dew point). However, these results do not occur consistently in a direction signifying heat stress under hot, humid conditions, so high CORT_B_ and a compromised stress response do not appear to be general responses of nighthawk chicks to potentially unfavorable thermal conditions on rooftops. As mentioned previously, chicks in the high CORT_B_ group did show a compromised ability to mount a stress response, and repeated extreme stochastic weather events predicted under future climate change scenarios (Meehl *et al.*, 2000) might have negative impacts on development or survival for at least some chicks.

We had predicted some associations between rooftop micro-climate variables and the CORT metrics, but found none. This suggests that rooftop micro-climate variables are not major effectors of baseline CORT or the stress response in nighthawk chicks or that sufficient variation among rooftops was not present to detect micro-climate effects. Perhaps this is due to the ability of the semi-precocial nighthawk chicks to seek favorable micro-climates (e.g. shady or windy sites) shortly after hatching. Their increased mobility relative to altricial species might alleviate some of the thermal load at the exposed nesting sites, and thus, chicks may be able to overcome some of the more extreme conditions reflected in the regional climate variables.

Corticosterone levels may vary in nestlings based upon their developmental strategy. For example, altricial nestlings, like barn owls, *Tyto alba* (Almansi *et al.*, 2009) and passerines (Tilgar *et al.*, 2017) have less developed hypothalamic–pituitary–adrenal (HPA) functionality at hatching and thus a lower stress response to poor environmental conditions, presumably due to allocation of resource investment to body growth. In contrast, chicks of semi-precocial species, such as Black-Legged Kittiwakes (*Rissa tridactyla*) (Kitaysky *et al.*, 2003), Red-legged Kittiwakes (*R. brevirostris*) (Kitaysky *et al.*, 2001), Tufted Puffins (*F. cirrhata*) (Kitaysky *et al.*, 2005), Ring-Billed Gulls (*L. delawarensis*) (Chin *et al.*, 2013), and Grey-faced Petrels (*Pterodroma macroptera gouldi*) (Adams *et al.*, 2008), have the capacity to mount a stress response. Because nighthawks are also semi-precocial (Brigham *et al.*, 2011), it might be expected that chicks are also capable of mounting a stress response. Indeed, the majority of nighthawk chicks in this study did mount a stress response to handling restraint. However, frequent exposure to extreme weather events can trigger a delayed emergency life history stage in which birds do not mount a stress response upon exposure to stressful conditions (Wingfield and Kitaysky, 2002). For a semi-precocial species that experiences frequent and unpredictable environmental perturbations, such as those at exposed rooftop nest sites of nighthawks, successive exposures to hot, humid conditions might elevate baseline CORT and reduce the capacity to mount a stress response to additional thermal challenges. For example, a stress response for a mobile second-week nestling that is left alone at night when the parents are feeding, might facilitate movement to find shelter from a thunderstorm, thereby prioritizing immediate survival over long-term growth. The low baseline CORT and capacity for stress responses in the majority of the chicks in our study, however, suggest that low metabolic cost alternatives, such micro-climate selection, gular fluttering, reduced activity and torpor (Lasiewski and Dawson, 1964; Brigham *et al.*, 1995; Fletcher *et al.*, 2004), rendered rooftop conditions as not thermally stressful to chicks.

A minority of chicks exposed to higher mean ambient temperature on the day prior to sampling, however, showed elevated baseline CORT, suggesting that the environment was stressful for these individuals. Stress responses to high temperatures in these chicks could be due to direct effects of temperature or to increased levels of dehydration resulting from increased evaporative cooling at high temperatures. Chicks did engage in gular flutter during laboratory studies at ambient temperatures above about 42°C and we also observed gular flutter in chicks on rooftops at high temperatures (Newberry and Swanson, unpubl. data). Because chicks do not have regular access to water on rooftops, high rates of evaporative cooling at high temperatures might be expected to result in dehydration, and dehydration (Arad and Skadhauge, 1984; Klingbeil, 1985) or hot, dry conditions (Treen *et al.*, 2015) may lead to increased baseline CORT in birds. Further study is needed to determine if the CORT responses to high temperatures documented for nighthawk chicks in this study result from direct effects of temperature, indirect effects of dehydration, or both.

While adult nighthawks and other nightjars in arid conditions are adaptable to extremely high temperatures (Cowles and Dawson, 1951; Howell, 1959; Bartholomew *et al.*, 1962; Lasiewski and Dawson, 1964; O’Connor *et al.*, 2017), increasing humidity in non-desert areas, such as our study sites, in the future (Kenward *et al.*, 2014) could negatively affect evaporative cooling capacity by lowering evaporative water loss rates in birds (Lasiewski *et al.*, 1966; Richards, 1976; Webster and King, 1987; Powers, 1992). Such a limitation on evaporative cooling might particularly impact chicks, with their less-developed capacities for evaporative cooling (Kirkham and Montevecchi, 1982; Speich and Manuwal, 1974). Low capacities for evaporative cooling might trigger elevated baseline corticosterone levels, which could potentially compromise growth, the immune system and survival of chicks (Sapolosky *et al.*, 2000; Love *et al.*, 2005). The chicks in the present study, however, did not show such a response to high humidities or high dew points, suggesting that current conditions of temperature and humidity at rooftop nest sites are not particularly stressful to nighthawk chicks. More research is needed to more fully quantify the relationships among nest site thermal conditions, and growth, survival and fitness for nighthawk chicks, which face challenges from both climate and land use change.

Some nighthawk chicks in the present study showed elevated baseline CORT associated with exposure to high ambient temperatures prior to blood sampling. The CORT-fitness hypothesis proposes that high baseline CORT can negatively impact survival or reproduction (Bonier *et al.*, 2009a) and although the hypothesis is controversial (e.g. Bonier *et al.*, 2009b; MacDougall-Shackleton *et al.*, 2009; Rivers *et al.*, 2012, 2017; Love *et al.*, 2014), some support for such a relationship exists for birds (e.g. Lanctot *et al.*, 2003; Buck *et al.*, 2007; Angelier *et al.*, 2010; Jaatinen *et al.*, 2013). If exposure to high temperatures routinely increases baseline CORT, either through direct temperature effects or indirect effects on dehydration, and high baseline CORT is negatively related to chick survival, this raises the possibility that future temperatures associated with climate change trends could make rooftops unsuitable for nighthawk nesting. If nighthawks are attracted to urban habitats as refugia in response to reductions of available natural habitats (Brigham *et al.*, 2011), increasing temperatures and the ‘urban heat island’ effect could render the rooftop nesting strategy as untenable. If so, nighthawks could be eliminated as a breeding species from this region and other similar regions where agricultural landscapes have reduced open natural habitats to low levels on the landscape.

## Supplementary Material

Supplementary Data
