# Supplementary material for: Elevated temperatures are associated with stress in rooftop-nesting Common Nighthawk (Chordeiles minor) chicks
Source: Conserv Physiol. 2018 Mar 1;6(1):coy010. doi: 10.1093/conphys/coy010 (PMC5830973; doi:10.1093/conphys/coy010)
Supplement: Supplementary Data [file newmicrosoftworddocument.docx]

**TABLE S1.** Summary of regression analyses of microclimate dependent variables with roof characteristics (as predictor variables). Dependent variables with *P*<0.05 at same direction of coefficient at all three weather stations are denoted with *. Dependent variables with *P*<0.05 at same direction of coefficient at two weather stations are denoted with **.

| **Predictor Variable** | **Dependent  Variable** | | ***F*** | | ***df*** | | **Adj *r^2^*** | | ***P*** | | ***Coef*** | |
| --- | --- | --- | --- | --- | --- | --- | --- | --- | --- | --- | --- | --- |
| Parapet Height (cm) | Anemometer T_a_ (°C) | 0.1093 | | 1,12 | | -0.074 | | 0.75 | | 0.0431 | |  |
|  | Anemometer Max Wind (KPH) | 2.291 | | 1,12 | | 0.090 | | 0.16 | | 1.7910 | |  |
|  | Ovoid T_e_ (°C) | 0.5369 | | 1,12 | | -0.037 | | 0.48 | | 0.1333 | |  |
|  | iButton T_a_ (°C) | 0.0239 | | 1,4 | | -0.243 | | 0.88 | | 0.0620 | |  |
| Roof Height (m) | Anemometer T_a_ (°C) | 0.0217 | | 1,12 | | -0.081 | | 0.89 | | -0.071 | |  |
|  | Anemometer Max Wind (KPH) | 0.3430 | | 1,12 | | -0.053 | | 0.57 | | -2.766 | |  |
|  | Ovoid T_e_ (°C) | 1.631 | | 1,12 | | 0.046 | | 0.23 | | 0.6972 | |  |
|  | iButton T_a_ (°C) | 23.59 | | 1,4  4 | | 0.819 | | <0.001** | | 1.6680 | |  |
|  | iButton T_a_ (°C) without outlier | 0.02426 | | 1,5 | | -0.1942 | | 0.88 | | 0.04733 | |  |
| Mean Gravel  Diameter (cm) | Anemometer T_a_ (°C) | 0.7808 | | 1,12 | | -0.017 | | 0.39 | | 0.3641 | |  |
|  | Anemometer Max Wind (KPH) | 0.0219 | | 1,12 | | -0.081 | | 0.89 | | 0.6184 | |  |
|  | Ovoid T_e_ (°C) | 0.0002 | | 1,12 | | -0.083 | | 0.99 | | 0.0062 | |  |
|  | iButton T_a_ (°C) | 0.0116 | | 1,4 | | -0.246 | | 0.92 | | -0.0943 | |  |

**TABLE S2:** Summary of regression analyses of log(CORT_B_/Mean+1), log(CORT_30_/Mean+1), and adjusted log(CORT_30_- CORT_B_/Mean+1) (as dependent variables) with temporal, roof, chick condition, microclimate and regional climate predictor variables of all chicks pooled. Dependent variables with *P*<0.05 are denoted with *.

| **Predictor Variable Model** | **Predictor Variable** | **Dependent  Variable** | | ***F*** | | ***df*** | | **Adj *r^2^*** | | ***P*** | | ***Coef*** |  |
| --- | --- | --- | --- | --- | --- | --- | --- | --- | --- | --- | --- | --- | --- |
| Temporal | Year | CORT_B_ | 0.1205 | | 3,20 | | -0.130 | | 0.65 | | -0.008 | | |
|  |  | CORT_30_ | 0.7386 | | 3,20 | | -0.035 | | 0.39 | | -6.696 | | |
|  |  | CORT_30_- CORT_B_ | 1.115 | | 3,20 | | 0.015 | | 0.23 | | -6.178 | | |
|  | Julian Date | CORT_B_ | 0.152 | | 1,22 | | -0.038 | | 0.70 | | -0.0031 | | |
|  |  | CORT_30_ | 1.93 | | 1,22 | | 0.039 | | 0.18 | | 0.0089 | | |
|  |  | CORT_30_- CORT_B_ | 1.115 | | 3,20 | | 0.015 | | 0.10 | | 0.0208 | | |
|  | Year and Julian Date | CORT_B_ | 0.894 | | 3,44 | | -0.007 | | 0.14 | | 0.0162 | | |
|  |  | CORT_30_ | 1.447 | | 3,20 | | 0.055 | | 0.17 | | 0.019 | | |
|  |  | CORT_30_- CORT_B_ | 2.139 | | 3,44 | | 0.068 | | 0.83 | | -0.001 | | |
|  | Time of Day | CORT_B_ | 0.1205 | | 3,20 | | -0.130 | | 0.65 | | -0.0021 | | |
|  |  | CORT_30_ | 0.7386 | | 3,20 | | -0.035 | | 0.79 | | -0.010 | | |
|  |  | CORT_30_- CORT_B_ | 1.115 | | 3,20 | | 0.015 | | 0.54 | | 0.0152 | | |
| Roof  Characteristics | Parapet Height  (cm) | CORT_B_ | 0.5254 | | 3,19 | | -0.069 | | 0.50 | | 0.0047 | | |
|  |  | CORT_30_ | 0.1854 | | 3,19 | | -0.125 | | 0.71 | | 0.0022 | | |
|  |  | CORT_30_- CORT_B_ | 0.9481 | | 3,19 | | -0.007 | | 0.47 | | -0.0025 | | |
|  | Roof Height  (m) | CORT_B_ | 0.5254 | | 3,19 | | -0.069 | | 0.81 | | 0.0071 | | |
|  |  | CORT_30_ | 0.1854 | | 3,19 | | -0.125 | | 0.81 | | 0.0061 | | |
|  |  | CORT_30_- CORT_B_ | 0.9481 | | 3,19 | | -0.007 | | 0.36 | | 0.0133 | | |
|  | Mean Gravel  Diameter (cm) | CORT_B_ | 0.5254 | | 3,19 | | -0.069 | | 0.36 | | 0.0240 | | |
|  |  | CORT_30_ | 0.1854 | | 3,19 | | -0.125 | | 0.56 | | 0.0131 | | |
|  |  | CORT_30_- CORT_B_ | 0.9481 | | 3,19 | | -0.007 | | 0.58 | | -0.0071 | | |
| Chick  Condition | Mass (g) | CORT_B_ | 0.0681 | | 2,21 | | -0.088 | | 0.99 | | 0.001 | | |
|  |  | CORT_30_ | 0.944 | | 2,21 | | -0.005 | | 0.27 | | 0.006 | | |
|  |  | CORT_30_- CORT_B_ | 2.266 | | 2,21 | | 0.099 | | 0.93 | | 0.001 | | |
|  | Wing Length  (cm) | CORT_B_ | 0.0681 | | 2,21 | | -0.088 | | 0.73 | | 0.002 | | |
|  |  | CORT_30_ | 0.944 | | 2,21 | | -0.005 | | 0.70 | | -0.001 | | |
|  |  | CORT_30_- CORT_B_ | 2.266 | | 2,21 | | 0.099 | | 0.06 | | -0.005 | | |
| Microclimate | Anemometer T_a_ (°C) | CORT_B_ | 0.193 | | 4,1 | | -1.822 | | 0.95 | | 1.059 | | |
|  |  | CORT_30_ | 0.2377 | | 4,1 | | -1.563 | | 0.91 | | 1.468 | | |
|  |  | CORT_30_- CORT_B_ | 0.6647 | | 4,1 | | -0.367 | | 0.58 | | -3.566 | | |
|  | Anemometer Max Wind (KPH) | CORT_B_ | 0.193 | | 4,1 | | -1.822 | | 0.92 | | 0.045 | | |
|  |  | CORT_30_ | 0.2377 | | 4,1 | | -1.563 | | 0.89 | | 0.048 | | |
|  |  | CORT_30_- CORT_B_ | 0.6647 | | 4,1 | | -0.367 | | 0.55 | | -0.107 | | |
|  | Ovoid T_e_ (°C) | CORT_B_ | 0.193 | | 4,1 | | -1.822 | | 0.93 | | -0.758 | | |
|  |  | CORT_30_ | 0.2377 | | 4,1 | | -1.563 | | 0.89 | | -0.940 | | |
|  |  | CORT_30_- CORT_B_ | 0.6647 | | 4,1 | | -0.367 | | 0.57 | | 2.068 | | |
|  | iButton T_a_ (°C) | CORT_B_ | 0.193 | | 4,1 | | -1.822 | | 0.65 | | 0.214 | | |
|  |  | CORT_30_ | 0.2377 | | 4,1 | | -1.563 | | 0.68 | | 0.144 | | |
|  |  | CORT_30_- CORT_B_ | 0.6647 | | 4,1 | | -0.367 | | 0.40 | | -0.168 | | |
| Regional Climate | Maximum T_a_ (°C) for Day | CORT_B_ | 2.371 | | 12,11 | | 0.417 | | 0.37 | | 0.1020 | | |
|  |  | CORT_30_ | 1.793 | | 12,11 | | 0.293 | | <0.05* | | 0.2293 | | |
|  |  | CORT_30_- CORT_B_ | 1.534 | | 12,11 | | 0.218 | | 0.08 | | 0.1351 | | |
|  | Maximum T_a_ (°C) for Week | CORT_B_ | 2.371 | | 12,11 | | 0.417 | | 0.42 | | 0.1589 | | |
|  |  | CORT_30_ | 1.793 | | 12,11 | | 0.293 | | 0.33 | | 0.1800 | | |
|  |  | CORT_30_- CORT_B_ | 1.534 | | 12,11 | | 0.218 | | 0.93 | | 0.0114 | | |
|  | Maximum Dew Point (°C) for Day | CORT_B_ | 2.371 | | 12,11 | | 0.417 | | <0.05* | | -0.3246 | | |
|  |  | CORT_30_ | 1.793 | | 12,11 | | 0.293 | | <0.05* | | -0.3103 | | |
|  |  | CORT_30_- CORT_B_ | 1.534 | | 12,11 | | 0.218 | | 0.77 | | -0.0217 | | |
|  | Maximum Dew Point (°C) for Week | CORT_B_ | 2.371 | | 12,11 | | 0.417 | | 0.21 | | -0.3468 | | |
|  |  | CORT_30_ | 1.793 | | 12,11 | | 0.293 | | 0.06 | | -0.5065 | | |
|  |  | CORT_30_- CORT_B_ | 1.534 | | 12,11 | | 0.218 | | 0.75 | | 0.0550 | | |
|  | Maximum Humidity ( %) for Day | CORT_B_ | 2.371 | | 12,11 | | 0.417 | | 0.12 | | 0.1854 | | |
|  |  | CORT_30_ | 1.793 | | 12,11 | | 0.293 | | 0.12 | | 0.1709 | | |
|  |  | CORT_30_- CORT_B_ | 1.534 | | 12,11 | | 0.218 | | 0.50 | | 0.0508 | | |
|  | Maximum Humidity ( %) for Week | CORT_B_ | 2.371 | | 12,11 | | 0.417 | | <0.05* | | -0.0388 | | |
|  |  | CORT_30_ | 1.793 | | 12,11 | | 0.293 | | 0.15 | | -0.2305 | | |
|  |  | CORT_30_- CORT_B_ | 1.534 | | 12,11 | | 0.218 | | 0.73 | | -0.0370 | | |
|  | Maximum Wind (KPH) for Day | CORT_B_ | 2.371 | | 12,11 | | 0.417 | | 0.92 | | -0.0032 | | |
|  |  | CORT_30_ | 1.793 | | 12,11 | | 0.293 | | 0.28 | | -0.0313 | | |
|  |  | CORT_30_- CORT_B_ | 1.534 | | 12,11 | | 0.218 | | 0.22 | | -0.0256 | | |
|  | Maximum Wind (KPH) for Week | CORT_B_ | 2.371 | | 12,11 | | 0.417 | | 0.15 | | 0.1596 | | |
|  |  | CORT_30_ | 1.793 | | 12,11 | | 0.293 | | 0.74 | | -0.0323 | | |
|  |  | CORT_30_- CORT_B_ | 1.534 | | 12,11 | | 0.218 | | <0.05* | | -0.1493 | | |
